# The “new-normal” OSCE examination: Executing in the COVID-19 era

**DOI:** 10.12669/pjms.37.7.4568

**Published:** 2021

**Authors:** Shazia Babar, Azam Afzal

**Affiliations:** 1 Shazia Babar Matin, Aga Khan University, Karachi, Pakistan; 2 Azam Saeed Afzal, Aga Khan University, Karachi, Pakistan

**Keywords:** OSCE, Face to Face, COVID-19

## Abstract

In this unprecedented situation of COVID-19 era, the educational institutions have to attune not only the teaching strategies but also the assessment. Similarly, just as COVID precautions have become the “new normal” practice, hence implementation changes during face-to-face examinations may become standard practice in the Post- COVID era. The Objective structured clinical exams (OSCEs) which usually require a face-to-face assessment of skills, posed a special challenge to health professionals in COVID 19. This commentary paper is written on shared experiences of the examination cell principal resource faculty for OSCE and exam coordinators. It addresses how to plan and implement objective, valid, feasible and reliable clinical skills examinations (OSCEs) keeping in place COVID precautions to ensure the utmost wellbeing of all stakeholders involved.

## BACKGROUND

The world has transformed as coronavirus spreads across the world. It is a universal health crisis; affecting all including the education sector.^1^ With the suspension of all face to face on-campus activities at institutions around the world due to the pandemic, everyone is forced to adapt to distance learning, in the form of online education.^1^ In this unprecedented situation, not only did the teaching strategies need to be attuned, but assessment had to be reformed too. Similarly, just as COVID precautions have become the “new normal” practice, hence implementation changes during face-to-face examinations may become standard practice in the Post- COVID era.

The COVID-19 pandemic has presented significant challenges for Health Care institutions.^1^ It is a demanding task to ensure that final year medical students graduate, and contribute in the healthcare work force in this time of crisis.^1^ It has also directed health care professionals towards hasty deviations in conducting assessments with a substantial shift to online assessment methods. Online assessments provide a good alternative to face-to-face written assessments for assessing knowledge. Adapting the assessment of skills online is proving to be much more challenging. During this ongoing pandemic, it is also empirical to assess and certify medical students’ for competencies that will be required from them in the new challenging healthcare work environment. Objective Structured Clinical Examinations (OSCEs) are assessments that measure clinical competence by physically rotating students through multiple stations. It requires considerable understanding of the essential educational philosophy of an OSCE with academic and administrative support for implemention.^2^,^3^ The Objective structured clinical exams (OSCEs) which usually require a face-to-face assessment of skills, posed a special challenge to health professionals in COVID-19. This commentary paper is written on shared experiences of the examination cell principal resource faculty for OSCE and exam coordinators of conducting OSCEs for more than a decade (90-100 summative exams cumulating close to 400-500 hours, and close to 2000 hours in exam analysis). It addresses how to plan and implement objective, valid, feasible and reliable clinical skills examinations (OSCEs) keeping in place COVID precautions to ensure the utmost wellbeing of all stakeholders involved.^4^

### Pre-OSCE Planning

In order to undertake this assessment, first and foremost a COVID free environment for the examination should be assured. For this, an appropriate examination venue should be identified. It should be adequately spacious such that the circuit permits each individual station to have adequate space (2 meters) for social distancing.^4^ The venue should preferably contain separate rooms for each station. If separate rooms are not available, then it should be ensured that appropriate distance is maintained between stations. It is also recommended to have separate exit and entry gates into the exam venue. The provision of hand sanitizer should be available at the examination venue entrance and exit, inside or outside every station and, and in student holding rooms, as well as ensuring that sanitizing sprays and appropriate cleaning materials are available at the venue. The examination venue must be re-cleaned on the morning of the OSCE with careful consideration of frequent touch points such as door handles prior to the start of the circuit. These safety measures were also mentioned by Katharine B et al in conducting OSCEs at Duke-National University Singapore Medical School.^1^

### Circuit Planning

When designing the OSCE circuit we need to consider the station task, and ordering of stations to curtail the risk to any stakeholders involved. Meticulous plan should be provided to invigilators and administrative staff regarding the movement to ensure students could be prevented from coming close to each other. In addition, it is recommended to keep the doors open while the examination is in progress and for smooth flow of the circuit to reduce spread of the disease through door handles.

### Human Resources

Experienced examiners should be engaged within the faculty. Examiners should be requested that if they have had COVID-19 symptoms in the seven days prior to the examination, or been exposed to a known COVID-19 positive individual in the previous 14 days, they must not attend and inform the exam organizers at the earliest convenience. Therefore, it is advisable to recruit additional back-up assessors in case any faculty tests positive for COVID or has to self-isolate as a precaution.

It is also advisable to recruit standardized patients (SPs) for the assessment. The SPs should be selected based on the case demographics and for better reliability of assessment they should be trained a day before the examination. Before training and the examination SPs should be screened and if required tested for COVID. Additional SPs should be recruited for any sudden last minute COVID screening failures and in case of non-availability of SPs; the examining faculty could take on the additional role of the SP. At Duke-NUS Medical School they recruited real patients for the OSCEs which was a challenge. They overcame this by utilizing very stable patients with chronic conditions, reducing the exchange of patients during the exam, and by screening them prior to the examination.^1^

Effective communication within the OSCE implementation team during the different phases of the process is essential to guarantee flawless planning and conduction of the assessment. To achieve effective communication, groups on messaging software such as WhatsApp can be created to facilitate discussion between the team members for timely management of emerging issues during planning.

### Prior to exam Day

A “dry” run of the OSCE circuit should be considered to test timing and sound equipment, and overall station setup. It is also necessary to have a pre-exam briefing for assessors, this may be done using a recorded briefing which can be sent to examiners in order to avoid a group briefing thus minimizing exposure. All of those participating on the day of the OSCE exam are recommended to use face shields & coverings, and should be reminded to maintain social distancing at all times.

Exam instructions should be emailed to students well before the exam. In addition, students should be briefed about the process of examination and reminded about the exam instructions and regulations. Ideally, the specific COVID-19 precautionary instructions should be emphasized, and students should be asked to repeat the instructions verbally back to the briefing organizer.

### Exam Day

At the exam venue entrance all participants must have their temperature checked on arrival. Ideally, students’ pre exam assembling area should be outdoors in an open space. Students must wait whilst SPs and examiners are directed straight to their stations. All assessors, students and SPs are required to wear face coverings and shields during the exam and requested to wash or sanitize their hands frequently.

### Post Exam

After the examination, students are allowed to leave first while assessors and SPs leave after so that everyone could maintain social distancing. Feedback from students, SPs and examiners about the exam is essential and can be done using anonymous online forms.

## CONCLUSION

The COVID-19 pandemic led to some specific challenges that needed to be overcome in order to deliver a face to face OSCE. The planning and mitigations put in place enabled this to occur with minimal risk to participants whilst delivering a defensible examination permitting students to be assessed. The COVID-19 pandemic has been a disruption to enable us to diversify the layout and delivery of teaching, learning and assessment. Most of these educational changes were initially born out of urgency, but many will likely remain in more refined forms as preferred methods of teaching and assessment in the future. Many lessons will be learnt during the months of this pandemic and educators will be better equipped to act promptly in future disruptions to medical education.^5^
